# SGB-ELM: An Advanced Stochastic Gradient Boosting-Based Ensemble Scheme for Extreme Learning Machine

**DOI:** 10.1155/2018/4058403

**Published:** 2018-06-26

**Authors:** Hua Guo, Jikui Wang, Wei Ao, Yulin He

**Affiliations:** ^1^School of Information Engineering, Lanzhou University of Finance and Economics, Lanzhou 730020, China; ^2^College of Computer Science & Software Engineering, Shenzhen University, Shenzhen 518060, China

## Abstract

A novel ensemble scheme for extreme learning machine (ELM), named Stochastic Gradient Boosting-based Extreme Learning Machine (SGB-ELM), is proposed in this paper. Instead of incorporating the stochastic gradient boosting method into ELM ensemble procedure primitively, SGB-ELM constructs a sequence of weak ELMs where each individual ELM is trained additively by optimizing the regularized objective. Specifically, we design an objective function based on the boosting mechanism where a regularization item is introduced simultaneously to alleviate overfitting. Then the derivation formula aimed at solving the output-layer weights of each weak ELM is determined using the second-order optimization. As the derivation formula is hard to be analytically calculated and the regularized objective tends to employ simple functions, we take the output-layer weights learned by the current pseudo residuals as an initial heuristic item and thus obtain the optimal output-layer weights by using the derivation formula to update the heuristic item iteratively. In comparison with several typical ELM ensemble methods, SGB-ELM achieves better generalization performance and predicted robustness, which demonstrates the feasibility and effectiveness of SGB-ELM.

## 1. Introduction

Extreme learning machine (ELM) was proposed as a promising learning algorithm for single-hidden-layer feedforward neural networks (SLFN) by Huang [1–3], which randomly chooses weights and biases for hidden nodes and analytically determines the output-layer weights by using Moore-Penrose (MP) generalized inverse [4]. Due to avoiding the iterative parameter adjustment and time-consuming weight updating, ELM obtains an extremely fast learning speed and thus attracts a lot of attention. However, random initialization of input-layer weights and hidden biases might generate some suboptimal parameters, which have negative impact on its generalization performance and predicted robustness.

To alleviate such weakness, many works have been proposed to further improve the generalization capability and stability of ELM, where ELM ensemble algorithms are the representative ones. Three representative ELM ensemble algorithms are summarized as follows. The earliest ensemble based ELM (EN-ELM) method was presented by Liu and Wang in [5]. EN-ELM introduced the cross-validation scheme into its training phase, where the original training dataset was partitioned into *R* subsets and then *R* pairs of training and validation sets were obtained so that each training set consists of *R*-1 subsets. Additionally, with updated input weights and hidden biases, *K* individual ELMs were trained based on each pair of the training and validation set. There were totally *K* × *R* ELMs that were constructed for decision-making in EN-ELM algorithm. Cao et al. [6] proposed a voting-based ELM (V-ELM) ensemble algorithm, which made the final decision based on the majority voting mechanism in classification applications. All the individual ELMs in V-ELM were trained on the same training dataset and the learning parameters of each basic ELM were randomly initialized independently. Moreover, a genetic ensemble of ELM (GE-ELM) method was designed by Xue et al. in [7], which used the genetic algorithm to produce optimal input weights as well as hidden biases for individual ELMs and selected ELMs equipped with not only higher fitness values but also smaller norm of output weights from the candidate networks. In GE-ELM, the fitness value of each individual ELM was evaluated based on the validation set which was randomly selected from the entire training dataset. There are still several other types of ELM ensemble algorithms which can be found in literatures [8–13].

As for ensemble of the traditional neural networks, the most prevailing approaches are Bagging and Boosting. In Bagging scheme [14], it generates several training datasets from the original training dataset and then trains a component neural network from each of those training datasets. Boosting mechanism [15] generates a series of component neural networks whose training datasets are determined by the performance of former ones. There are also many other approaches for training the component neural networks. Hampshire [16] utilizes different object functions to train distinct component neural networks. Xu et al. [17] introduce the stochastic gradient boosting ensemble scheme to bioinformatics applications. Yao et al. [18] regard all the individuals in an evolved population of neural networks as component networks.

In this paper, a new ELM ensemble scheme called Stochastic Gradient Boosting-based Extreme Learning Machine (SGB-ELM) which makes use of the mechanism of stochastic gradient boosting [19, 20] is proposed. SGB-ELM constructs an ensemble model by training a sequence of ELMs where the output weights of each individual ELM is learned by optimizing the regularized objective in an additive manner. More specifically, we design an objective based on the training mechanism of boosting method. In order to alleviate overfitting, we introduce a regularization item which controls the complexity of our ensemble model to the objective function concurrently. Then the derivation formula aimed at solving output weights of the newly added ELM is determined by optimizing the objective using second-order approximation. As the output weights of the newly added ELM at each iteration are hard to be analytically calculated based on the derivation formula, we take the output weights learned by the pseudo-residuals-based training dataset as an initial heuristic item and thus obtain the optimal output weights by using the derivation formula to update the heuristic item iteratively. Because the regularized objective tends to employ not only predictive but also simple functions and meanwhile a randomly selected subset rather than the whole training set is used to minimize training residuals at each iteration, SGB-ELM can continually improve the generalization capability of ELM while effectively avoiding overfitting. The experimental results in comparison with Bagging ELM, Boosting ELM, EN-ELM, and V-ELM show that SGB-ELM obtains better classification and regression performances, which demonstrates the feasibility and effectiveness of SGB-ELM algorithm.

The rest of this paper is organized as follows. In Section 2, we briefly summarize the basic ELM model as well as the stochastic gradient boosting method.  Section 3 introduces our proposed SGB-ELM algorithm. Experimental results are presented in Section 4. Finally, we conclude this paper and make some discussions in Section 5.

## 2. Preliminaries

In this section, we briefly review the principles of basic ELM model and the stochastic gradient boosting method to provide necessary backgrounds for the development of SGB-ELM algorithm in Section 3.

### 2.1. Extreme Learning Machine

ELM is a special learning algorithm for SLFN, which randomly selects weights (linking the input layer to the hidden layer) and biases for hidden nodes and analytically determines the output weights (linking the hidden layer to the output layer) by using MP generalized inverse. Suppose we have a training dataset with *N* instances *𝔻* = {(x_*i*_, y_*i*_)}_*i*=1_^*N*^, where x_i_ = (*x*_*i*1_, *x*_*i*2_, ⋯, *x*_*id*_) ∈ R^*d*^ and y_*i*_ = (*y*_*i*1_, *y*_*i*2_, ⋯, *y*_*im*_) ∈ R^*m*^. It is known that *m* = 1 for regression and *m* > 1 for classification. In ELM, the input weights and hidden biases can be randomly chosen according to* any continuous probability distribution* [2]. Namely, we randomly select the learning parameters within the range of [−1,1] as

(1) $$\begin{array}{c} W={\left[\;\begin{array}{c} w_{1}\\ w_{2}\\ \vdots \\ w_{L} \end{array}\;\right]}^{T}={\left[\;\begin{array}{cccc} w_{11} & w_{12} & \cdots & w_{1L}\\ w_{21} & w_{22} & \cdots & w_{2L}\\ \vdots & \vdots & \ddots & \vdots \\ w_{d1} & w_{d2} & \cdots & w_{dL} \end{array}\;\right]}_{d\times L} \end{array}$$

and

(2) $$\begin{array}{c} B={\left[\;b_{1},b_{2},\cdots ,b_{L}\;\right]}^{T}, \end{array}$$

where *L* is the number of hidden-layer nodes in SLFN. Depending on the theory proved in [2], the output-layer weights in ELM model can be analytically calculated by

(3) $$\begin{array}{c} \beta =H^{†}Y. \end{array}$$

Here, H^†^ is the MP generalized inverse of the hidden-layer output matrix

(4) $$\begin{array}{c} H={\left[\;g\left(\;x_{i}w_{l}+b_{l}\;\right)\;\right]}_{N\times L}, \end{array}$$

where *i* = 1,2, ⋯, *N*,  *l* = 1,2, ⋯, *L*, and *g*(*u*) = 1/(1 + exp⁡(−*u*)) is the sigmoid activation function, and

(5) $$\begin{array}{c} Y=\left[\begin{array}{c} y_{1}\\ y_{2}\\ \vdots \\ y_{N} \end{array}\right]={\left[\;\begin{array}{cccc} y_{11} & y_{12} & \cdots & y_{1m}\\ y_{21} & y_{22} & \cdots & y_{2m}\\ \vdots & \vdots & \ddots & \vdots \\ y_{N1} & y_{N2} & \cdots & y_{Nm} \end{array}\;\right]}_{N\times m} \end{array}$$

is the target matrix. Generally, for an unseen instance $\hat{x}=\left({\hat{x}}_{1},{\hat{x}}_{2},\cdots ,{\hat{x}}_{d}\right)$, ELM predicts its output $\hat{y}$ as follows:

(6) $$\begin{array}{c} \hat{y}=h\left(\;\hat{x}\;\right)\beta , \end{array}$$

where $h\left(\hat{x}\right)=\left[g\left(\hat{x}w_{1}+b_{1}\right),\cdots ,g\left(\hat{x}w_{L}+b_{L}\right)\right]$ is the hidden-layer output vector of $\hat{x}$.

Due to avoiding the iterative adjustment to input-layer weights and hidden biases, ELM's training speed can be thousands of times faster than those of traditional gradient-based learning algorithms [2]. At the meantime, ELM also produces good generalization performance. It has been verified that ELM can achieve the equal generalization performance with the typical Support Vector Machine algorithm [3].

### 2.2. Stochastic Gradient Boosting

Stochastic gradient boosting scheme was proposed by Friedman in [20], and it is a variant of the gradient boosting method presented in [19]. Given a training set {(x_*i*_, y_*i*_)}_*i*=1_^*N*^, the goal is to learn a hypothesis *F*_*K*_(x) that maps x to y and minimizes the training loss as follows:

(7) $$\begin{array}{c} F_{K}\left(\;x\;\right)=\underset{F_{K}\left(x\right)}{arg\;min}\overset{N}{\underset{i=1}{\sum }}L\left(\;y_{i},F_{K}\left(\;x_{i}\;\right)\;\right), \end{array}$$

where *L*(·, ·) is the loss function which evaluates the difference between the predicted value and the target and K denotes the number of iterations. In boosting mechanism, K additive individual learners are trained sequentially by

(8) $$\begin{array}{c} f_{k}\left(\;x\;\right)=\underset{f_{k}\left(x\right)}{arg\;min}\overset{N}{\underset{i=1}{\sum }}L\left(\;y_{i},F_{k-1}\left(\;x_{i}\;\right)+f_{k}\left(\;x_{i}\;\right)\;\right) \end{array}$$

and

(9) $$\begin{array}{c} F_{k}\left(\;x\;\right)=F_{k-1}\left(\;x\;\right)+f_{k}\left(\;x\;\right), \end{array}$$

where *k* = 1,2, ⋯, *K*. It is shown that the optimization problem depends much on the loss function and becomes unsolvable when *L*(·, ·) is complex. Creatively, gradient boosting constructs the weak individuals based on the pseudo residuals, which are the gradient of loss function with respect to the model values predicted at the current learning step. For instance, let **ϵ**_*i*_^(*k*)^ be the pseudo residual of the *i*th sample at the *k*th iteration written as

(10) $$\begin{array}{c} \epsilon_{i}^{\left(k\right)}=-{\left[\;\frac{\partial L\left(y_{i},\hat{y}\right)}{\partial \hat{y}}\;\right]}_{\hat{y}=F_{k-1}\left(x_{i}\right)}, \end{array}$$

and thus the *k*th weak learner *E*_*k*_(x) is trained by

(11) $$\begin{array}{c} E_{k}\left(\;x\;\right)=\underset{E_{k}\left(x\right)}{arg\;min}\overset{N}{\underset{i=1}{\sum }}L\left(\;\epsilon_{i}^{\left(k\right)},E_{k}\left(\;x_{i}\;\right)\;\right). \end{array}$$

As gradient boosting constructs additive ensemble model by sequentially fitting a weak individual learner to the current pseudo-residuals of whole training dataset at each iteration, it costs much training time and may suffer from overfitting problem. In view of that, a minor modification named stochastic gradient boosting is proposed to incorporate some randomization to the procedure. Specifically, at each iteration a randomly selected subset instead of the full training dataset is used to fit the individual learner and compute the model update for the current iteration. Namely, let {*π*(*i*)}_1_^*N*^ be a random permutation of the integers {1,2, ⋯, *N*}, and the subset with size $\tilde{N}<N$ of the entire training dataset can be given by ${\left\{\left(x_{\pi \left(i\right)},y_{\pi \left(i\right)}\right)\right\}}_{i=1}^{\tilde{N}}$. Furthermore, the *k*th weak learner using the stochastic gradient boosting ensemble scheme is trained by solving the following optimization problem as

(12) $$\begin{array}{c} E_{k}^{*}\left(\;x\;\right)=\underset{E_{k}^{*}\left(x\right)}{arg\;min}\overset{\tilde{N}}{\underset{i=1}{\sum }}L\left(\;\epsilon_{\pi \left(i\right)}^{\left(k\right)},E_{k}^{*}\left(\;x_{\pi \left(i\right)}\;\right)\;\right). \end{array}$$

Given the base learner *F*_0_(x) which is trained by the initial training dataset, the final ensemble learning model constructed by stochastic gradient boosting scheme predicts an unknown testing instance $\hat{x}$ as follows:

(13) $$\begin{array}{c} F_{K}\left(\;\hat{x}\;\right)=F_{0}\left(\;\hat{x}\;\right)+\overset{K}{\underset{k=1}{\sum }}E_{k}^{*}\left(\;\hat{x}\;\right). \end{array}$$

Stochastic gradient boosting is also considered as a special linear search optimization algorithm, which makes the newly added individual learner fit the fastest descent direction of partial training loss at each learning step.

## 3. Stochastic Gradient Boosting-Based Extreme Learning Machine (SGB-ELM)

SGB-ELM is a novel hybrid learning algorithm, which introduces the stochastic gradient boosting method into ELM ensemble procedure. As boosting mechanism focuses on gradually reducing the training residuals at each iteration and ELM is a special multiparameters network (for classification tasks particularly), instead of combining the ELM and stochastic gradient boosting primitively, we design an enhanced training scheme to alleviate possible overfitting in our proposed SGB-ELM algorithm. The detailed implementation of SGB-ELM is presented in Algorithm 2, where the determination of optimal output weights for each individual ELM learner is illustrated in Algorithm 1 accordingly.

There are many existing second-order approximation methods including sequential quadratic programming (SQP) [21] and majorization-minimization algorithm (MM) [22]. SQP is an effective method for nonlinearly constrained optimization by solving quadratic subproblems. MM aims to optimize the local alternative objective which is easier to solve in comparison with the original cost function. Instead of using second-order approximation directly, SGB-ELM designs an optimization criterion for the output-layer weights of each individual ELM. In view of that, quadratic approximation is merely employed as an optimization tool in SGB-ELM.

In SGB-ELM, the key issue is to determine the optimal output-layer weights of each weak individual ELM, which is expected to further decrease the training loss and meanwhile keep a simple network structure. Consequently, we design a learning objective considering not only the fitting ability for training instances but also the complexity of our ensemble model as follows:

(14) $$\begin{array}{c} Q^{\left(K\right)}=\overset{N}{\underset{i=1}{\sum }}L\left(\;y_{i},{\hat{y}}_{i}\;\right)+\lambda \overset{K}{\underset{k=1}{\sum }}\Omega \left(\;E_{k}\;\right), \end{array}$$

where *L*(·, ·) is a differentiable loss function that measures the difference between the predicted output ${\hat{y}}_{i}$ and the target value y_*i*_. The second term *Ω* represents the complexity of the ensemble model consisting of *K* weak individual learners. Moreover, *λ* is a regularization factor that makes a balance between training loss and architectural risk. It is obvious that the objective falls back to the traditional gradient booting method when the regularization factor *λ* is set to zero.

As for boosting training mechanism, each individual ELM is greedily added to the current ensemble model sequentially so that it can most improve our model according to (8). Specifically, let ${\hat{y}}_{i}^{\left(k-1\right)}$ be the predicted value of the *i*th instance at the (*k* − 1)th iteration and *E*_*k*_(x) be the *k*th weak ELM learner that needs to be incorporated into the ensemble model, then the prediction of the *i*th instance at the *k*th iteration ${\hat{y}}_{i}^{\left(k\right)}$ can be written as

(15) $$\begin{array}{c} {\hat{y}}_{i}^{\left(k\right)}={\hat{y}}_{i}^{\left(k-1\right)}+E_{k}\left(\;x_{i}\;\right),\;k=1,2,\cdots ,K. \end{array}$$

In order to obtain the newly added individual ELM, we first introduce *E*_*k*_(x) to the existing learned ensemble model and then minimize the following objective:

(16) Qk=∑i=1NLyi,y^ik+λ∑j=1kΩEj=∑i=1NLyi,y^ik−1+Ekxi+λ∑j=1k−1ΩEj+ΩEk,

where *E*_1_, *E*_2_, ⋯, *E*_*k*−1_ is already obtained at the previous iterations. As a consequence, the complexity of the learned ensemble model ∑_*j*=1_^*k*−1^*Ω*(*E*_*j*_) is a constant, and we only need to take *Ω*(*E*_*k*_) into consideration. Removing the constant item, the objective *Q*^(*k*)^ is simplified as

(17) $$\begin{array}{c} Q^{\left(k\right)}=\overset{N}{\underset{i=1}{\sum }}L\left[\;y_{i},{\hat{y}}_{i}^{\left(k-1\right)}+E_{k}\left(\;x_{i}\;\right)\;\right]+\lambda \Omega \left(\;E_{k}\;\right). \end{array}$$

Stochastic gradient boosting selects a random subset with size $\tilde{N}<N$ of the whole training set to fit the individual learner at each iteration. Namely, let {*π*(*i*)}_1_^*N*^ be a random permutation of the integers {1,2, ⋯, *N*}, then we can define a stochastic subset as ${\left\{\left(x_{\pi \left(i\right)},y_{\pi \left(i\right)}\right)\right\}}_{i=1}^{\tilde{N}}$. Accordingly, the objective using stochastic gradient boosting is transformed as

(18) $$\begin{array}{c} Q^{\left(k\right)}=\overset{\tilde{N}}{\underset{i=1}{\sum }}L\left[\;y_{\pi \left(i\right)},{\hat{y}}_{\pi \left(i\right)}^{\left(k-1\right)}+E_{k}\left(\;x_{\pi \left(i\right)}\;\right)\;\right]+\lambda \Omega \left(\;E_{k}\;\right). \end{array}$$

We use second-order approximation to optimize the above learning objective, where the lose function is derived by Taylor expansion as follows:

(19) Lk≃Lyj,y^jk−1+ujkTEkxj+12vjkTEk2xj,

where *j* = *π*(*i*) is the new index for (x_*π*(*i*)_, y_*π*(*i*)_) in the randomly generated subset,

(20) $$\begin{array}{c} u_{j}^{\left(k\right)}=\left[\;{\left.\;\frac{\partial L\left(y_{\pi \left(i\right)},\hat{y}\right)}{\partial \hat{y}}\;\right|}_{\hat{y}=F_{k-1}\left(x_{\pi \left(i\right)}\right)}\;\right],\;i=1,2,\cdots ,\tilde{N} \end{array}$$

is the first-order gradient statistics on the loss function with respect to the current predicted output *F*_*k*−1_(x_*π*(*i*)_), and

(21) $$\begin{array}{c} v_{j}^{\left(k\right)}=\left[\;{\left.\;\frac{\partial L^{2}\left(y_{\pi \left(i\right)},\hat{y}\right)}{\partial {\hat{y}}^{2}}\;\right|}_{\hat{y}=F_{k-1}\left(x_{\pi \left(i\right)}\right)}\;\right],\;i=1,2,\cdots ,\tilde{N} \end{array}$$

is the second-order gradient statistics on the loss function with respect to the current predicted output *F*_*k*−1_(x_*π*(*i*)_). Due to the approximation for training loss, we can provide a general solution scheme regardless of the specific type of loss function. In addition, second-order optimization tends to achieve better convergence in comparison with the traditional gradient method [23]. Obviously, $L\left[y_{j},{\hat{y}}_{j}^{\left(k-1\right)}\right]$ is a fixed value, and thus the objective can be further expressed as

(22) Qk=∑j=1N~ujkTEkxj+12vjkTEk2xj+λΩEk.

Let $\left[𝒳;𝒴\right]={\left\{\left(x_{\pi \left(i\right)},y_{\pi \left(i\right)}\right)\right\}}_{i=1}^{\tilde{N}}$, and the objective can be rewritten in a matrix form as

(23) Qk=TrUkTEkX+12TrVkTEk2X+λΩEk,

where

(24) $$\begin{array}{c} U_{k}=\left[\begin{array}{c} u_{\pi \left(1\right)}^{\left(k\right)}\\ u_{\pi \left(2\right)}^{\left(k\right)}\\ \vdots \\ u_{\pi \left(\tilde{N}\right)}^{\left(k\right)} \end{array}\right]={\left[\;\begin{array}{cccc} u_{11}^{\left(k\right)} & u_{12}^{\left(k\right)} & \cdots & u_{1m}^{\left(k\right)}\\ u_{21}^{\left(k\right)} & u_{22}^{\left(k\right)} & \cdots & u_{2m}^{\left(k\right)}\\ \vdots & \vdots & \ddots & \vdots \\ u_{\tilde{N}1}^{\left(k\right)} & u_{\tilde{N}2}^{\left(k\right)} & \cdots & u_{\tilde{N}m}^{\left(k\right)} \end{array}\;\right]}_{\tilde{N}\times m} \end{array}$$

and

(25) $$\begin{array}{c} V_{k}=\left[\begin{array}{c} v_{\pi \left(1\right)}^{\left(k\right)}\\ v_{\pi \left(2\right)}^{\left(k\right)}\\ \vdots \\ v_{\pi \left(\tilde{N}\right)}^{\left(k\right)} \end{array}\right]={\left[\;\begin{array}{cccc} v_{11}^{\left(k\right)} & v_{12}^{\left(k\right)} & \cdots & v_{1m}^{\left(k\right)}\\ v_{21}^{\left(k\right)} & v_{22}^{\left(k\right)} & \cdots & v_{2m}^{\left(k\right)}\\ \vdots & \vdots & \ddots & \vdots \\ v_{\tilde{N}1}^{\left(k\right)} & v_{\tilde{N}2}^{\left(k\right)} & \cdots & v_{\tilde{N}m}^{\left(k\right)} \end{array}\;\right]}_{\tilde{N}\times m}. \end{array}$$

The *k*th individual learner *E*_*k*_(*𝒳*) is a basic ELM model, which randomly selects input-layer weights W_*k*_ and hidden biases B_*k*_. Given the hidden-layer output matrix

(26) $$\begin{array}{c} H_{k}=g\left(\;XW_{k}+B_{k}\;\right)={\left[\;\begin{array}{cccc} h_{11}^{\left(k\right)} & h_{12}^{\left(k\right)} & \cdots & h_{1L}^{\left(k\right)}\\ h_{21}^{\left(k\right)} & h_{22}^{\left(k\right)} & \cdots & h_{2L}^{\left(k\right)}\\ \vdots & \vdots & \ddots & \vdots \\ h_{\tilde{N}1}^{\left(k\right)} & h_{\tilde{N}2}^{\left(k\right)} & \cdots & h_{\tilde{N}L}^{\left(k\right)} \end{array}\;\right]}_{\tilde{N}\times L}, \end{array}$$

*E*_*k*_(*𝒳*) can be expressed as

(27) $$\begin{array}{c} E_{k}\left(\;X\;\right)=H_{k}\beta_{k}, \end{array}$$

where

(28) $$\begin{array}{c} \beta_{k}={\left[\;\begin{array}{cccc} \beta_{11}^{\left(k\right)} & \beta_{12}^{\left(k\right)} & \cdots & \beta_{1m}^{\left(k\right)}\\ \beta_{21}^{\left(k\right)} & \beta_{22}^{\left(k\right)} & \cdots & \beta_{2m}^{\left(k\right)}\\ \vdots & \vdots & \ddots & \vdots \\ \beta_{L1}^{\left(k\right)} & \beta_{L2}^{\left(k\right)} & \cdots & \beta_{Lm}^{\left(k\right)} \end{array}\;\right]}_{L\times m} \end{array}$$

is the output-layer weight matrix that needs to be determined. As Bartlett [24] pointed out that networks tend to perform better generalization with not only small training error but also small norm of weights (‖**β**_*k*_‖), we use L2-norm to evaluate the complexity of a basic ELM model as

(29) $$\begin{array}{c} \Omega \left(\;E_{k}\;\right)=\frac{1}{2}{\left\|\;\beta_{k}\;\right\|}^{2}. \end{array}$$

Accordingly, the conclusive objective can be written as

(30) Qk=TrUkTHkβk+12TrVkTHkβk2+λ2βk2.

From (30), we can find that the objective is only sensitive to **β**_*k*_ at the *k*th iteration. For single-variable optimization, solving partial derivative is conducted as

(31) $$\begin{array}{c} \frac{\partial Q^{\left(k\right)}}{\partial \beta_{k}}=0, \end{array}$$

where each element in **β**_*k*_ conducted a partial derivative, respectively. Thus we obtain the derivation formula as follows:

(32) $$\begin{array}{c} \beta_{lj}^{\left(k\right)}=-\frac{\sum_{i=1}^{\tilde{N}}\left[u_{ij}^{\left(k\right)}h_{il}^{\left(k\right)}+v_{ij}^{\left(k\right)}h_{il}^{\left(k\right)}\sum_{s=1,s\neq l}^{L}h_{is}^{\left(k\right)}\beta_{sj}^{\left(k\right)}\right]}{\sum_{i=1}^{\tilde{N}}v_{ij}^{\left(k\right)}{\left[h_{il}^{\left(k\right)}\right]}^{2}+\lambda \tilde{N}}, \end{array}$$

where *l* = 1,2, ⋯, *L* and *j* = 1,2, ⋯, *m*. It is shown that **β**_*k*_ is difficult to be calculated analytically. Since our designed regularized objective tends to generate an ensemble model employing predictive as well as simple hypotheses, (32) derived by the objective can be used as an optimization criterion. Specifically, we take the output-layer weights determined by pseudo-residuals dataset as an initial heuristic item and thus obtain the optimal output-layer weights by using the derivation formula to update the heuristic item iteratively. Algorithm 1 illustrates how the optimal output weight matrix is determined and the detailed implementation of SGB-ELM is presented in Algorithm 2.

In Algorithm 2, all the input weights and hidden biases of individual ELMs are randomly chosen within the range of [−1,1]. For boosting-based ensemble methods, the initial base learner is expected to be enhanced by adding weak individual learners to the current ensemble model step by step. In view of that, high-precision initial base learner might affect the effectiveness of ensemble negatively. In order to control the fitting ability of the initial base learner *F*_0_(x) and meanwhile reduce the instability brought by random determination of the input weights and hidden biases, SGB-ELM conducts multiple random initializations for parameters in ELM_0_ and takes the average at last. For instance, we take the average of 100 random initializations as

(33) wil=∑t=1100wilt100,bl=∑t=1100blt100,

where *i* = 1,2, ⋯, *d* and *l* = 1,2, ⋯, *L*. For the weak individual ELM, which plays a smaller role in the whole ensemble model, random initialization of parameters exactly increases the diversity between weak individual learners.

## 4. Performance Validation

In this section, a series of experiments are conducted to validate the feasibility and effectiveness of our proposed SGB-ELM algorithm, and meanwhile we compare the generalization performance and predicted stability of several typical ensemble learning methods (EN-ELM [5], V-ELM [6], Bagging [14], and Adaboost [15]) on 4 KEEL [25] regression and 5 UCI [26] classification datasets. Among all the above-mentioned ensemble methods, the basic ELM model proposed in [2] is used as the individual learner, where the sigmoid function *g*(*x*) = 1/(1 + exp⁡(−*x*)) is selected as the activation function. All the experiments are carried out on a desktop computer with Win10 operating system, Intel (R) i5-4590 3.30GHz CPU, and 12GB memory and implemented with Matlab 9.0 version. Meanwhile, all the experimental results are the average of 50 repeated trials. The experiments are generally divided into two parts: one part is to evaluate the performance of SGB-ELM, and the other part is to measure the effect of learning parameters on training SGB-ELM algorithm.

### 4.1. Performance Evaluation of SGB-ELM

For regression problem, the performances of SGB-ELM and other comparative algorithms are both measured by Root Mean Square Error (RMSE), which reveals the difference between the predicted value and the target. Additionally, in this paper, we take the squared loss as our loss function in SGB-ELM algorithm for regression task. Suppose ${\hat{y}}_{i}$ and *y*_*i*_ are the predicted value and the target of the *i*th instance, respectively, and the loss function *L*(·, ·) is given by

(34) $$\begin{array}{c} L\left(\;y_{i},{\hat{y}}_{i}\;\right)=\frac{1}{2}{\left(\;{\hat{y}}_{i}-y_{i}\;\right)}^{2}. \end{array}$$

Since V-ELM and EN-ELM are designed for classification applications, we compare the generalization capability of SGB-ELM with the basic ELM, simple ensemble ELM, Bagging ELM, and Adaboost ELM in regression tasks. Among them, simple ensemble ELM can be considered as a variant of the V-ELM method, which trains a number of individual ELMs independently and takes the simple average of all the predictions at last. Adaboost ELM is implemented by Adaboost.R2 method [27], which applies the primitive Adaboost algorithm designed for classification tasks [15] to the regression field. Furthermore, we adopt resampling the original training dataset rather than assigning a weight to every instance to train each individual learner in Adaboost.R2 ELM.

**Figure 1:**
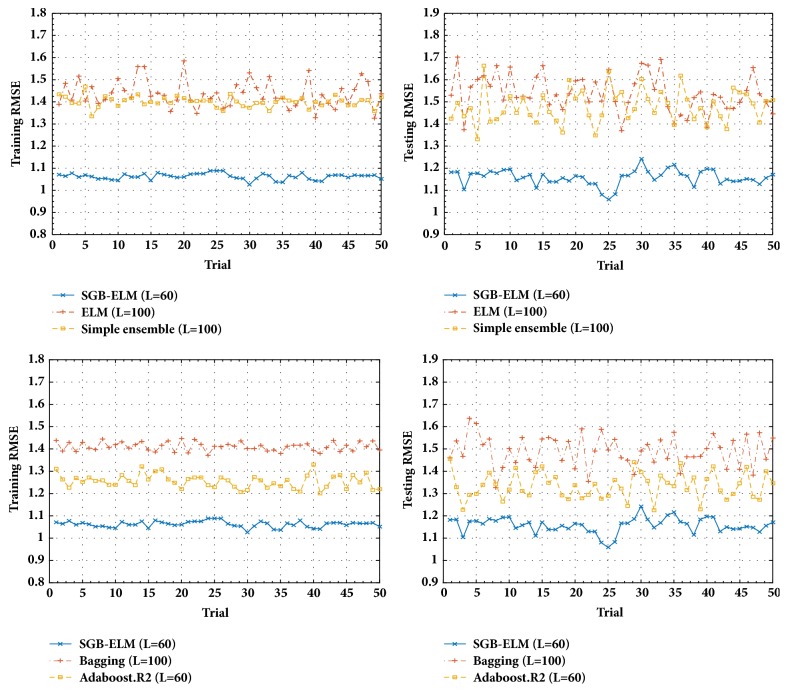
The training and testing performance of ELM, simple ensemble, Bagging, Adaboost.R2, and SGB-ELM during 50 trials on the Friedman dataset.

The performances of the traditional ELM, simple ensemble ELM, Bagging ELM, Adaboost ELM, and our proposed SGB-ELM are compared on 4 representative regression datasets, which are selected from the KEEL [25] repository. Experimentally, all the inputs of each dataset are normalized into the range of [0,1]. The characteristics of these datasets are summarized in Table 1, where each original dataset is divided into two groups including a training set (70%) and a testing set (30%). In our regression experiments, for each dataset, the number of hidden nodes *L* is selected from {10,20, ⋯, 200}. The parameters in SGB-ELM are set as *λ* = {0.001,0.0015, ⋯, 0.01},  $\tilde{N}/N=\left\{0.1,0.2,\cdots ,1\right\}$, and *K* = 50. The settings of other comparative algorithms can be found in Table 2.  Figure 1 shows the training and testing RMSE of different learning methods during 50 trials on Friedman dataset. The detailed comparison results between SGB-ELM and other learning algorithms on 4 regression benchmark datasets are shown in Table 2. Furthermore, we compare the training and testing performances of SGB-ELM with those of Adaboost.R2 with regard to the number of iterations on Mortgage dataset, which is presented in Figure 3(a).

As for classification problem, like other typical feedforward neural networks (for instance, BP neural networks [28]), SGB-ELM evaluates the predicted output by calculating the sum of squared errors. Specifically, let ${\hat{y}}_{i}$ be the predicted output vector and y_*i*_ be the target encoded by One-Hot scheme [29] of the *i*th sample, respectively, and we define the loss function *L*(·, ·) in SGB-ELM for classification as follows:

(35) $$\begin{array}{c} L\left(\;y_{i},{\hat{y}}_{i}\;\right)=\frac{1}{2}{\left\|\;{\hat{y}}_{i}-y_{i}\;\right\|}^{2}=\frac{1}{2}\overset{m}{\underset{j=1}{\sum }}{\left(\;{\hat{y}}_{ij}-y_{ij}\;\right)}^{2}. \end{array}$$

It is shown that SGB-ELM aims at reducing the training RMSE inch by inch for classification problem. Accordingly, we compare SGB-ELM with several representative ensemble learning methods including V-ELM, EN-ELM, Bagging ELM, and Adaboost ELM. Among them, V-ELM and EN-ELM have been briefly summarized in*Section 1*, and Adaboost ELM is implemented by Adaboost.SAMME method [30], which extends the original Adaboost designed for binary classification to multiclassification problem.

**Figure 2:**
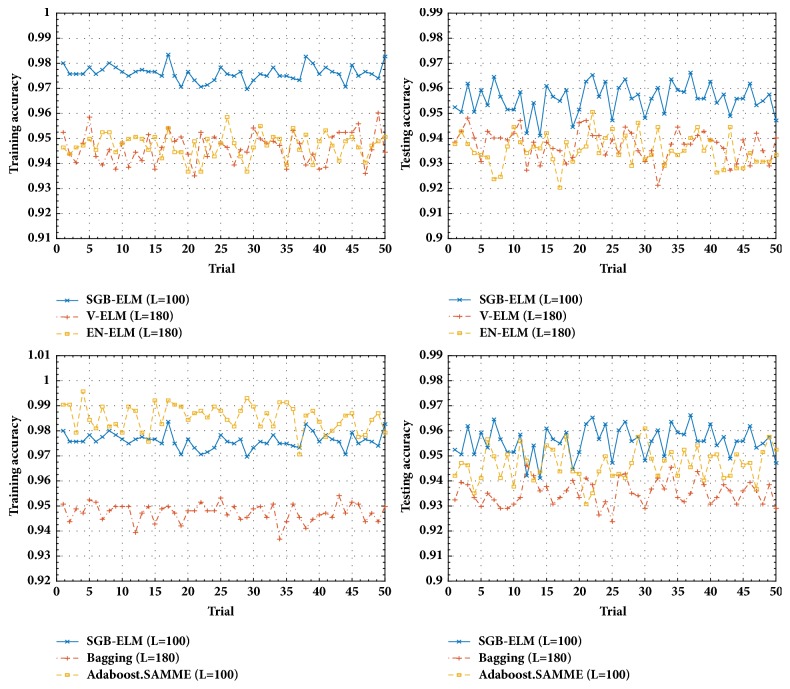
The training and testing performance of V-ELM, EN-ELM, Bagging, Adaboost.SAMME, and SGB-ELM during 50 trials on the Segmentation dataset.

**Figure 3:**
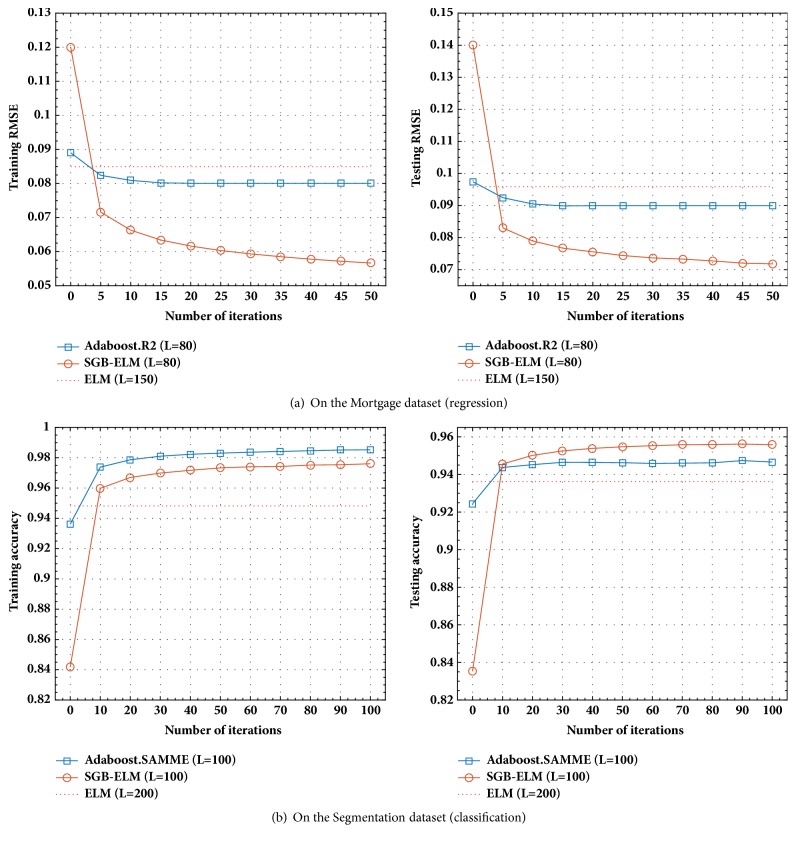
The training and testing RMSE/accuracy of SGB-ELM with regard to the number of iterations in comparison with Adaboost method.

Similarly, we select 5 popular classification datasets from the UCI Machine Learning Repository [26] to verify the performance of our proposed SGB-ELM algorithm. For each dataset, all the decision attributes are encoded by One-Hot scheme [29]. The characteristics of these datasets are described in Table 3, where each original data set is equally divided into two groups including a training set (50%) and a testing set (50%). The number of hidden nodes is also selected from *L* = {10,20, ⋯, 200} for each dataset. The parameters in SGB-ELM are set as *λ* = {0.001,0.002, ⋯, 0.03},  $\tilde{N}/N=\left\{0.1,0.2,\cdots ,1\right\}$, and *K* = 100. The cross-validation is tenfold (*R* = 10) in EN-ELM. The number of individual ELMs for ensemble is 7 (*K* = 7) in V-ELM. Other settings can be found in Table 4.  Figure 2 shows the training and testing accuracy of different algorithms during 50 trials on Segmentation dataset. The detailed performances of SGB-ELM in comparison with other learning algorithms on 5 classification benchmark datasets are summarized in Table 4. Lastly, the training and testing accuracy of SGB-ELM and Adaboost.SAMME with regard to the number of iterations on the Segmentation dataset are presented in Figure 3(b).

Tables 2 and 4 present the comparison results including training time, training RMSE/accuracy, and testing RMSE/accuracy for regression and classification tasks, respectively. It is shown that SGB-ELM obtains the better generalization capability in most cases without significantly increasing the training time. At the same time, SGB-ELM tends to have smaller training Dev and testing Dev than those of the comparative learning algorithms, which exactly validates the robustness and stability of our proposed SGB-ELM Algorithm. In particular, since SGB-ELM adopts the similar training mechanism with Adaboost which integrates multiple weak individual learners sequentially, the number of hidden nodes is set as a smaller value in both SGB-ELM and Adaboost method. It is worth noting that SGB-ELM can achieve better performance than the existing methods with less hidden nodes and outperforms Adaboost with the same number of hidden nodes.

From Figures 1 and 2, we can find that SGB-ELM is more stable than the traditional ELM, simple ensemble, Bagging, and Adaboost.R2 in regression problem and also produces better robustness than V-ELM, EN-ELM, Bagging, and Adaboost.SAMME in classification problem. It is shown that SGB-ELM not only focuses on reducing the predicted bias as other boosting like methods, but also generates a robust ensemble model with a low variance. As observed in Figure 2 although Adaboost.SAMME generates higher training accuracy than SGB-ELM during the most of 50 trials, SGB-ELM obtains the better generalization capability (testing accuracy). It can be explained by two reasons as (1) we introduce a regularization item (L2-norm) to the learning objective to control the complexity of our ensemble learning model; (2) a randomly selected subset rather than the whole training dataset is used to minimize the training loss at each iteration in our proposed SGB-ELM algorithm.

Figure 3 shows the training RMSE/accuracy and testing RMSE/accuracy of Adaboost (Adaboost.R2 for regression and Adaboost.SAMME for classification) and SGB-ELM with regard to the number of iterations. The fixed reference line denotes the training and testing performance of a traditional ELM, which is equipped with much more hidden nodes. As shown in Figure 3, SGB-ELM obviously improves the generalization capability of the initial base ELM in both regression and classification tasks. From Figure 3(a), we can find that the training and testing RMSE is declining gradually as the number of iterations increases. Similarly, both the training and testing accuracy curve show an increasing trend in Figure 3(b). Because we conduct multiple random initializations for parameters in the initial base learner *F*_0_ and take the average at last, the fitting ability of *F*_0_ is artificially weakened to some extent. As a result, the initial training and testing RMSE/accuracy of SGB-ELM are much lower than the initial Adaboost. It is shown that both SGB-ELM and Adaboost outperform the traditional ELM equipped more hidden nodes after a small number of learning steps. Furthermore, we can find that SGB-ELM produces better performance than Adaboost after only 5 iterations in regression tasks and 10 iterations in classification tasks. It verifies the significant convergence of second-order optimization method, which is incorporated into the procedure of SGB-ELM.

From the experimental results of both regression and classification problems, we can conclude that our proposed SGB-ELM algorithm can not only achieve better generalization capability (low predicted bias) than the typical existing variants of ELM, but also obtain an enough robust ELM ensemble learning model (low predicted variance).

### 4.2. Impact of Learning Parameters on Training SGB-ELM

To achieve good generalization performance, three learning parameters of SGB-ELM including the number of hidden nodes *L*, the regularization factor *λ*, and the size of subset $\tilde{N}$ need to be chosen appropriately. In this section, we attempt to evaluate the impact of learning parameters on training SGB-ELM algorithm and provide some empirical references of choosing these parameters.

**Figure 4:**
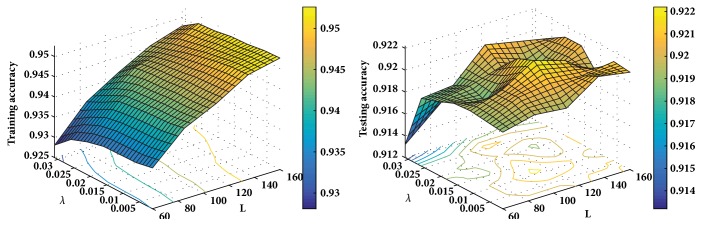
The training and testing performance of SGB-ELM with different combinations of (*L*, *λ*) on the Spambase dataset.

**Figure 5:**
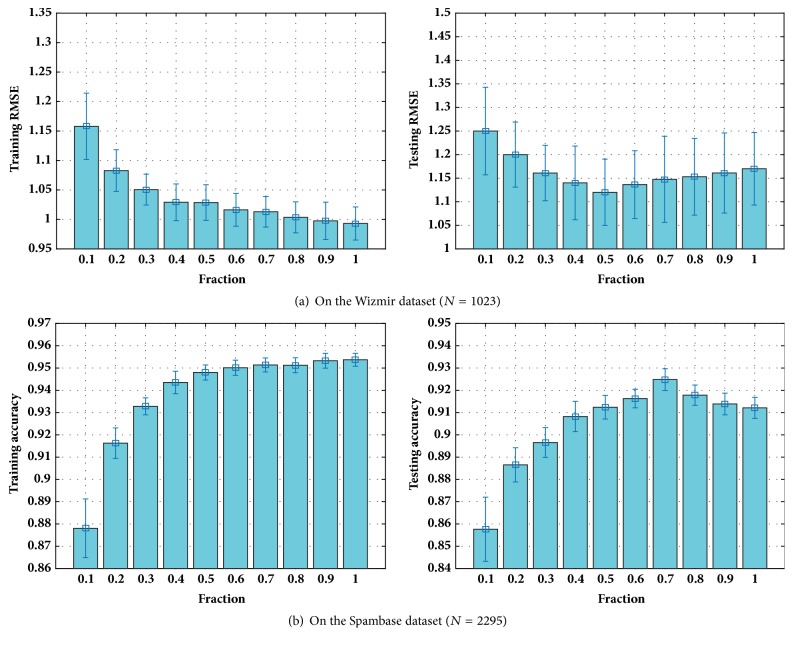
The training and testing performance of SGB-ELM with different sampling fraction ($\tilde{N}/N$) on the Wizmir and Spambase datasets.

For the basic ELM model, the number of hidden nodes *L* decides the model's capacity. In other words, an ELM with more hidden nodes is more complex and can deal with more training instances. However, it tends to obtain an overfitting model when *L* is set as a value too large. The regularization factor *λ* makes a balance between the training loss and the complexity of model. It means that *λ* can control the capacity or the complexity of our model. The size of subset $\tilde{N}$ represents the number of training instances at each iteration and it introduces some randomization to the training procedure of SGB-ELM. Firstly, we use grid-search method to observe the training and testing performance of SGB-ELM with different *L* and *λ*. Specifically, we set *L* = {10,15, ⋯, 160},  *λ* = {0.001,0.002, ⋯, 0.03}, and a fixed $\tilde{N}=0.8$. The training and testing performance of SGB-ELM with regard to the combination of (*L*, *λ*) on the Spambase dataset is shown in Figure 4. Secondly, as we empirically find that the optimal $\tilde{N}$ depends much on the size of training dataset, we conduct two experiments (including a small dataset and a large dataset) to measure the impact of $\tilde{N}$ on training SGB-ELM. We choose the optimal value of (*L*, *λ*) according to the grid-search results and set $\tilde{N}/N=\left\{0.1,0.2,\cdots ,1\right\}$.  Figure 5 shows the training and testing performance of SGB-ELM with different sampling fraction ($\tilde{N}/N$) on the Wizmir and Spambase datasets.

As shown in Figure 4, changing the value of (*L*, *λ*) has a significant effect on the training and testing accuracy of SGB-ELM algorithm. It is obvious that SGB-ELM with excess hidden nodes is more likely to produce overfitting when the regularization factor *λ* is set as a small value. It also demonstrates that SGB-ELM can effectively reduce overfitting when *λ* is assigned a proper value. In addition, from Figure 4 we can find that SGB-ELM achieves better performance with enough hidden nodes and a proper *λ*. It can be explained by the rule that although SGB-ELM with a small number of hidden nodes can avoid overfitting intuitively, meanwhile it produces a barrier to fit the current training residuals appropriately.

From Figure 5, it is obvious that randomization improves the performance of SGB-ELM substantially. As each weak individual ELM is learned based on randomly selected subset of the whole training dataset, it exactly increases the diversity between all the individuals. On the other hand, randomization introduces a noisy estimate of the total training loss. As a result, it slows down the convergence and even makes the learning curve fluctuate (higher variance) if $\tilde{N}$ is too small. It is shown that the best value of the sampling fraction is approximately 50% on the Wizmir dataset and 70% on the Spambase dataset, where there are a typical improvement in testing performance comparing to no sampling at all. Since the optimal values of $\tilde{N}/N$ are different on the Wizmir and Spambase datasets, it indicates that the sampling fraction ($\tilde{N}/N$) is expected to be determined based on the specific learning tasks and assigned a bigger value on the training dataset containing more instances.

## 5. Conclusions

In this paper, we proposed a novel ensemble model named Stochastic Gradient Boosting-based Extreme Learning Machine (SGB-ELM). Instead of combining ELM and stochastic gradient boosting primitively, we construct an ELM flow or ELM sequence where the output-layer weights of each weak ELM are determined by optimizing the regularized objective additively. Firstly, by minimizing the objective using second-order approximation, the derivation formula aimed at solving the output-layer weights of each individual ELM is determined. Then we take the output-layer weights learned by the current pseudo residuals as a heuristic item and thus obtain the optimal output-layer weights by updating the heuristic item iteratively. The performance of SGB-ELM was evaluated on 4 regression and 5 classification datasets. In comparison with several typical ELM ensemble methods, SGB-ELM obtained better performance and robustness, which demonstrated the feasibility and effectiveness of SGB-ELM algorithm.

## Figures and Tables

**Algorithm 1:**
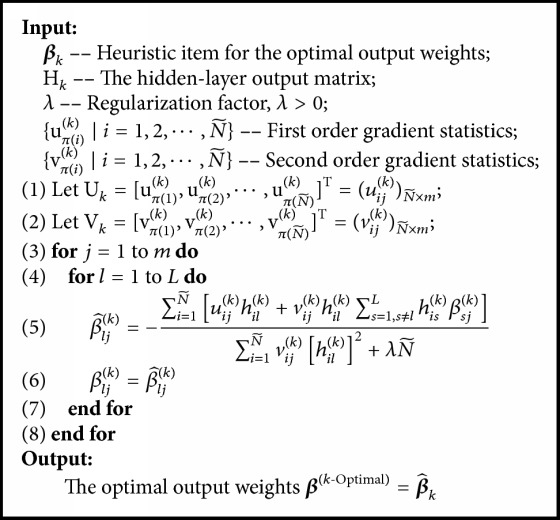
The determination of **β**^(*k*-Optimal)^.

**Algorithm 2:**
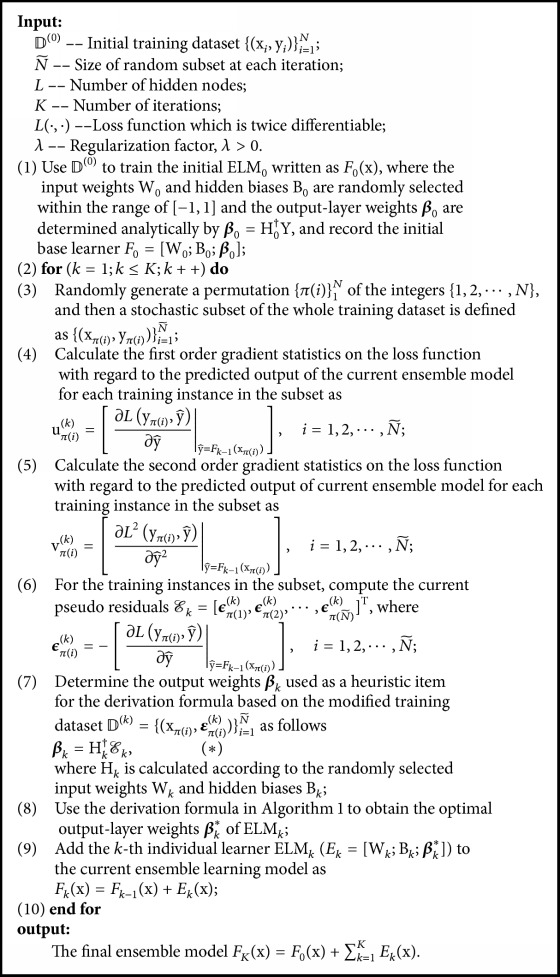
SGB-ELM.

**Table 1:** Details of 4 KEEL regression datasets.

** No. **	** Datasets **	** Condition attributes **	** Training samples **	** Testing samples **
1	Laser	4	695	298
2	Friedman	5	840	360
3	Mortgage	15	734	315
4	Wizmir	9	1023	438

**Table 2:** The comparison results between SGB-ELM and other representative algorithms on 4 regression datasets.

**Dataset **	**Algorithm **	**Training time **	**Training RMSE (Dev) **	**Testing RMSE (Dev) **	**Hidden nodes **	**Iterations **
Laser ($\tilde{N}/N=0.4$)	ELM	0.0097	12.3791 ± 0.6067	12.7783 ± 1.5863	80	N/A
	Simple ensemble	0.1547	12.1216 ± 0.4585	12.8794 ± 1.5415	80	10
	Bagging	0.7991	12.1707 ± 0.5094	13.4085 ± 1.5998	80	50
	Adaboost	0.1591	11.4460 ± 0.4875	12.0666 ± 1.2080	50	Max = 50
	SGB-ELM	3.4853	7.6354 ± 0.3919	**8.4170 **±** 1.0788**	50 (*λ* = 0.001)	50

Friedman ($\tilde{N}/N=0.5$)	ELM	0.0141	1.4220 ± 0.0532	1.5124 ± 0.0906	100	N/A
	Simple ensemble	0.2081	1.4005 ± 0.0240	1.4791 ± 0.0747	100	10
	Bagging	1.0144	1.4111 ± 0.0197	1.4906 ± 0.0701	100	50
	Adaboost	0.5219	1.2551 ± 0.0304	1.3342 ± 0.0587	60	Max = 50
	SGB-ELM	4.8853	1.0627 ± 0.0136	**1.1581 **±** 0.0346**	60 (*λ* = 0.002)	50

Mortgage ($\tilde{N}/N=0.4$)	ELM	0.0200	0.0855 ± 0.0022	0.0961 ± 0.0078	150	N/A
	Simple ensemble	0.3044	0.0843 ± 0.0021	0.0947 ± 0.0085	150	10
	Bagging	1.4544	0.0834 ± 0.0018	0.0937 ± 0.0077	150	50
	Adaboost	0.4778	0.0785 ± 0.0020	0.0885 ± 0.0058	80	Max = 50
	SGB-ELM	6.2434	0.0607 ± 0.0015	**0.0759 **±** 0.0056**	80 (*λ* = 0.004)	50

Wizmir ($\tilde{N}/N=0.5$)	ELM	0.0128	1.0906 ± 0.0277	1.1263 ± 0.0667	100	N/A
	Simple ensemble	0.2066	1.0869 ± 0.0269	1.1203 ± 0.0629	100	10
	Bagging	1.0366	1.0859 ± 0.0259	1.1165 ± 0.0620	100	50
	Adaboost	0.4331	1.0622 ± 0.0519	1.1091 ± 0.0857	60	Max = 50
	SGB-ELM	5.6525	1.0148 ± 0.0258	**1.1032 **±** 0.0615**	60 (*λ* = 0.002)	50

**Table 3:** Details of 5 UCI classification datasets.

** No. **	** Datasets **	** Condition attributes **	** Decision attributes **	** Training samples **	** Testing samples **
1	Image segmentation	19	7	1155	1155
2	Texture	40	11	2750	2750
3	Spambase	57	2	2295	2294
4	Banana	2	2	2650	2650
5	Ring	20	2	3700	3700

**Table 4:** The comparison results between SGB-ELM and other representative algorithms on 5 classification datasets.

**Dataset **	**Algorithm **	**Training time **	**Training accuracy (Dev) **	**Testing accuracy (Dev) **	**Hidden nodes **	**Iterations **
Segmentation ($\tilde{N}/N=0.5$)	ELM	0.0431	0.9465 ± 0.0055	0.9351 ± 0.0064	180	N/A
	V-ELM	0.4487	0.9463 ± 0.0061	0.9374 ± 0.0060	180	7
	EN-ELM	43.9234	0.9472 ± 0.0048	0.9353 ± 0.0063	180 (*R* = 10)	50
	Bagging	3.1853	0.9474 ± 0.0035	0.9353 ± 0.0050	180	50
	Adaboost	3.5372	0.9853 ± 0.0052	0.9466 ± 0.0067	100	Max = 100
	SGB-ELM	134.2969	0.9761 ± 0.0030	**0.9558 **±** 0.0049**	100 (*λ* = 0.004)	100

Texture ($\tilde{N}/N=0.7$)	ELM	0.0338	0.9954 ± 0.0011	0.9945 ± 0.0016	100	N/A
	V-ELM	0.4275	0.9965 ± 0.0007	0.9950 ± 0.0013	100	7
	EN-ELM	44.4969	0.9963 ± 0.0008	0.9946 ± 0.0011	100 (*R* = 10)	50
	Bagging	3.0959	0.9965 ± 0.0007	0.9957 ± 0.0013	100	50
	Adaboost	10.3628	0.9996 ± 0.0017	0.9972 ± 0.0024	60	Max = 100
	SGB-ELM	193.2019	0.9992 ± 0.0005	**0.9982 **±** 0.0008**	60 (*λ* = 0.002)	100

Spambase ($\tilde{N}/N=0.7$)	ELM	0.0459	0.9174 ± 0.0044	0.9080 ± 0.0061	150	N/A
	V-ELM	0.4213	0.9192 ± 0.0042	0.9115 ± 0.0053	150	7
	EN-ELM	62.6000	0.9183 ± 0.0054	0.9071 ± 0.0060	150 (*R* = 10)	50
	Bagging	2.9869	0.9219 ± 0.0039	0.9145 ± 0.0051	150	50
	Adaboost	7.6875	0.9620 ± 0.0046	**0.9234 **± 0.0072	100	Max = 100
	SGB-ELM	129.0922	0.9522 ± 0.0033	0.9222 ±** 0.0043**	100 (*λ* = 0.006)	100

Banana ($\tilde{N}/N=0.6$)	ELM	0.0550	0.6838 ± 0.0253	0.6787 ± 0.0263	180	N/A
	V-ELM	0.4906	0.6860 ± 0.0261	0.6848 ± 0.0264	180	7
	EN-ELM	67.5578	0.6821 ± 0.0227	0.6780 ± 0.0250	180 (*R* = 10)	50
	Bagging	3.3253	0.6808 ± 0.0164	0.6777 ± 0.0178	180	50
	Adaboost	7.5100	0.7457 ± 0.0288	0.7448 ± 0.0320	100	Max = 100
	SGB-ELM	133.0791	0.7610 ± 0.0085	**0.7563 **±** 0.0082**	100 (*λ* = 0.004)	100

Ring ($\tilde{N}/N=0.8$)	ELM	0.0897	0.9492 ± 0.0024	0.9418 ± 0.0035	200	N/A
	V-ELM	0.7609	0.9532 ± 0.0024	0.9466 ± 0.0032	200	7
	EN-ELM	114.0641	0.9517 ± 0.0028	0.9418 ± 0.0031	200 (*R* = 10)	50
	Bagging	5.5241	0.9539 ± 0.0027	0.9468 ± 0.0030	200	50
	Adaboost	17.2109	0.9940 ± 0.0023	0.9524 ± 0.0038	150	Max = 100
	SGB-ELM	363.7976	0.9750 ± 0.0021	**0.9567 **±** 0.0027**	150 (*λ* = 0.008)	100
